# Feeding and resting behaviours of natural *Anopheles gambiae* s.l. populations in an area of low malaria transmission in south-western Senegal: A comparative study between mainland and island settings

**DOI:** 10.1186/s13071-025-07230-y

**Published:** 2026-01-14

**Authors:** Moussa Diop, Youssouph Coulibaly, Cheikh Lo, Abdoulaye Kane Dia, Ndeye Aita Ndoye, Edouard Guedj Tine, Ndeye Seny Diagne, Yaya Ibrahim Coulibaly, Modibo Sangaré, Omar Thiaw, Mouhamadou Bassir Faye, Mame Fatou Tall, Oumar Ciss, Ousmane Faye, Abdoulaye Niang, Lassana Konaté, Neil F. Lobo, Roger Clément Kouly Tine, El Hadji Amadou Niang

**Affiliations:** 1 Laboratoire d’Ecologie Vectorielle et Parasitaire, Département de Biologie Animale, Faculté des Sciences et Techniques, Université Cheikh Anta Diop de Dakar, BP 5005, Dakar, Sénégal; 2 Mali International Center for Excellence in Research (ICER), Techniques, and Technologies of Bamako (USTTB), University of Sciences, Bamako, Mali; 3 Eck Institute for Global Health, University of Notre Dame, Notre Dame, IN 46556 USA; 4 Service de Parasitologie Et Mycologie, Faculté de Médecine, Pharmacie Et Odontostomatologie, Université Cheikh Anta Diop de Dakar, Dakar, Sénégal

**Keywords:** Feeding, Resting behaviour, *Anopheles gambiae*, Malaria transmission, Mainland, Island, South-western, Senegal

## Abstract

**Background:**

The biting and resting behaviours of *Anopheles* species, which are human malaria vectors, are specifically linked to ecological and climatic requirements that characterize certain geographical settings, such as forests and humid savannah areas where favourable conditions for malaria mosquitoes are found. In southern Senegal, the outdoor resting behaviour of *Anopheles gambiae* s.l. populations is suspected to be a major problem in malaria control, given that indoor-based control tools are currently deployed across the country. A longitudinal study was conducted to investigate the population dynamics, trophic preferences and resting behaviours of *Anopheles gambiae* s.l. in mainland and island areas in south-western Senegal.

**Methods:**

Indoor and outdoor resting mosquitoes were collected from September 2020 to November 2021 using Pyrethrum Spray Catches and Prokopack aspirators, respectively. Field-collected mosquitoes were morphologically identified using conventional dichotomous keys, and in the laboratory, the mosquito blood meal source and molecular species identification were determined using enzyme-linked immunosorbent assays and polymerase chain reaction, respectively.

**Results:**

Out of 765 *Anopheles* collected, 181 were from the mainland, and 584 were from the island. *Anopheles gambiae* s.l. was the predominant species (91.1%), with seasonal variation. The indoor resting densities did not significantly differ (*P* = 0.082) between the mainland (0.41 females per room) and island (4.09 females per room) areas. In mainland areas, the human blood index (HBI) was significantly greater (*P* = 0.035) in indoor resting females (76.2%) than in outdoor resting females (47.6%), whereas in island areas, the HBI was generally lower, with no significant difference (*P* = 0.51) between indoor (25.1%) and outdoor (31.1%) resting populations. Endophilic populations had greater HBIs in mainland areas than in island areas (*P* = 2.63 × 10^−5^), whereas no significant difference was observed for exophilic populations (*P* = 0.13).

**Conclusions:**

These findings provide a preliminary basic understanding of the feeding and resting behaviours of *Anopheles gambiae* s.l. populations in mainland and island areas for evidence-based malaria control programmes.

**Graphical Abstract:**

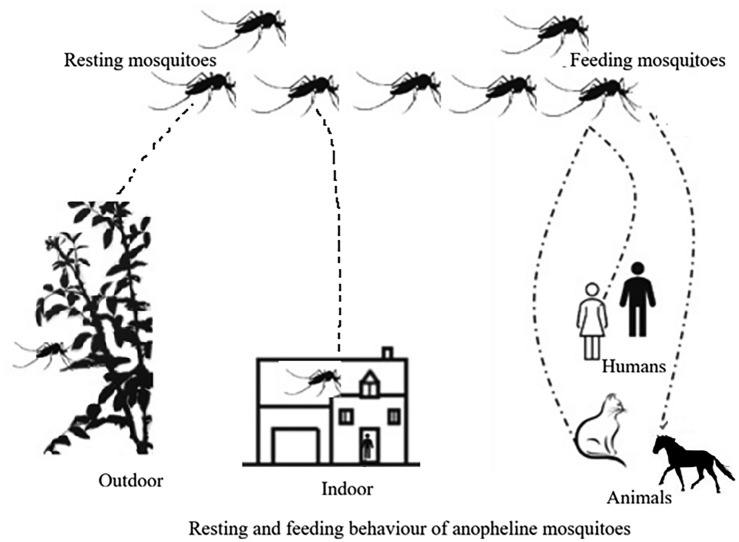

## Background

Several *Anopheles* species are vectors of infectious diseases, such as malaria, which is among the major public health problems in sub-Saharan Africa [1]. In the western African region, malaria incidence is particularly high in humid savannah and forests, where vectors find favourable conditions for their development and survival [2, 3]. In Senegal, more than 20 anopheline species have been described thus far, including at least seven known malaria vectors. Of these, *Anopheles gambiae* s.s., *Anopheles coluzzii*, *Anopheles arabiensis* [4, 5] and *Anopheles funestus* play a primary role in malaria transmission, while the remaining species, such as *Anopheles. nili*, *Anopheles melas* and *Anopheles pharoensis,* are secondary vectors [6–9]. Each of these vector species has its own bionomic traits, including resting behaviour, which govern species adaptation to vector control interventions.

Current core malaria vector interventions include indoor residual spraying (IRS) and long-lasting-insecticide-treated nets (LLINs) [10], which primarily target indoor resting (endophilic) and indoor biting (endophagic) anopheline populations [1]. Indoor residual spraying can be very effective for endophilic vector populations that are susceptible to the insecticide formulated for spraying purposes. Over the past two decades, IRS and LLINs have contributed to averting almost 70% of malaria cases worldwide [11]. Despite the effectiveness of these interventions, several studies have demonstrated that malaria elimination goals are jeopardized by residual transmission sustained by outdoor biting and outdoor resting vectors [12, 13]. Understanding vector resting behaviour is fundamental before implementing IRS at a given site. Vector resting behaviour is also an important factor when evaluating the impact of IRS, thus enabling possible shifts to be pinpointed as adaptive responses to control tools. Indoor resting mosquitoes are usually collected using the pyrethrum spray catches (PSC) method, which has several limitations, mainly because of its inability to obtain samples of outdoor resting specimens. To fill this gap, the Prokopack aspirator has often been used simultaneously with PSCs.

In Senegal, the coverage rates of LLINs and IRS in the southern part of the country in 2022 reached 99% and 94.6%, respectively. Thus, these strategies have significantly contributed to reducing malaria incidence in this region [14]. In addition to their effectiveness, the widespread implementation of IRS and LLINs has consequently induced evolutionary adaptations in malaria vector populations, leading to resistance to or escape from these insecticide-based interventions. Major adaptations include insecticide resistance and shifts in biting and resting behaviours [15–17]. To maintain the effectiveness of current vector control tools and reduce protection gaps, more targeted control measures are needed. This study investigated the feeding and resting behaviours of *An. gambiae* s.l. populations from mainland and island areas in south-western Senegal.

## Methods

### Study area

**Fig. 1:**
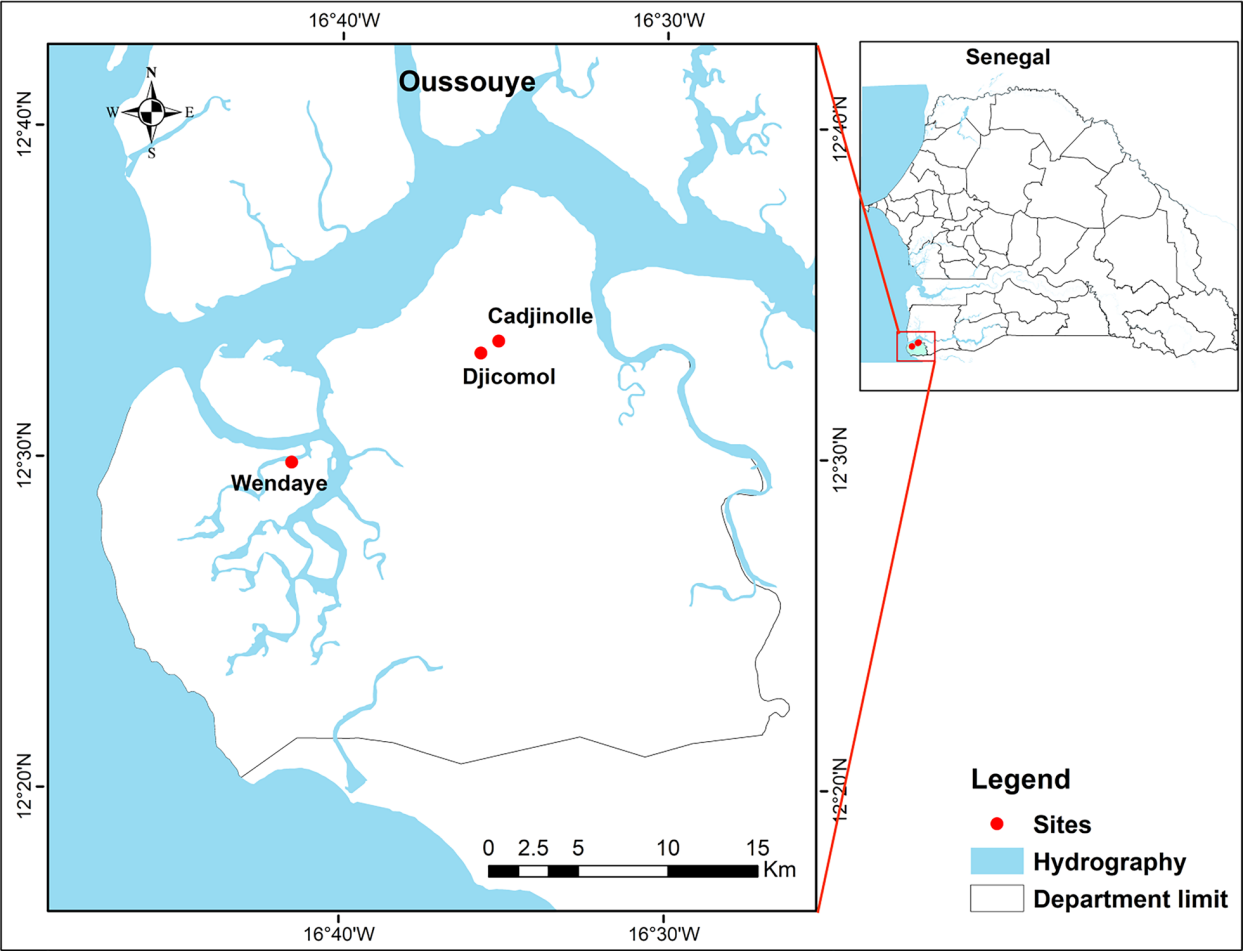
Location of the study sites

A longitudinal study was conducted in south-western Senegal from September 2020 to November 2021 in two different ecological settings (mainland and island). Two neighbouring sites, Djicomol (12°33′11″N, 16°35′43″W) and Cadjinolle (12°33′33″N, 16°34′70″W), located in the village of Mlomp, were selected in the mainland area. In the island area, Wendaye (12°29′51″N;,16°41′31″W), which is in the Diembering heath district and one of the numerous insular villages within the specific Bolong ecosystem (swampy brackish water river system), was selected. Mlomp and Diembering are located in Oussouye, one of the three departments of the Ziguinchor region (Fig. 1). The village of Mlomp, which covers an area of 70 km^2^, is located approximately 500 km from Dakar, the capital city of Senegal, 50 km west of the city of Ziguinchor and 25 km north of the border with Guinea-Bissau [18]. The health district of Diembering, covering 237 km^2^, is located alongside the coast of the Atlantic Ocean. It is bounded towards the north by the Casamance River, to the east by the communes of Mlomp and Oukout, to the south by the Republic of Guinea-Bissau and to the west by the Atlantic Ocean. The Casamance River and various tributaries provide a Diembering hydrographic network, which has created, in the deltaic area, the Bolong’s swampy brackish water river ecosystem, specific to the Casamance Natural Region and its multitudes and scattered insular villages. Wendaye, as with other islands, is characterized by the density and extent of the hydrographic network running deep into the communes. All villages are either adjacent to riverine backwaters or have a river running through them [19]. The climate of the study area is of the Sudano-Guinean type, with a rainy season lasting from mid-June to November and a dry season from December to mid-June. The annual rainfall is relatively high, estimated between 1200 and 2000 mm. The temperatures are generally moderate, ranging from 15 to 18 °C during the cold season and from 27 to 32 °C during the hot season [20]. The major activity in the southern part of the country is rice growing, along with microgardening, which generates substantial anopheline larval breeding sites.

### Mosquito sampling and laboratory processing

Indoor and outdoor resting mosquitoes were sampled monthly in 2020 and bimonthly in 2021 using pyrethrum spray catches (PSCs) and Prokopack aspirators, respectively. Pyrethrum spray catches were carried out from 7 a.m. to 9 a.m. inside ten randomly selected rooms in each study village. In the meantime, collections with the Prokopack aspirator were conducted for approximately 10 min, targeting all potential outdoor shelters within and around the ten concessions selected for PSCs, within a radius of up to 50 m centred on each surveyed household. Upon collection, the mosquitoes were sorted to the genus level, and *Anopheles* species were identified using conventional keys [6]. The collected resting females were further classified according to their abdomen state, i.e., unfed, fed, half-gravid and gravid females. All the sampled anopheline specimens were individually stored in numbered Eppendorf tubes containing desiccant and kept for subsequent laboratory processing.

In the laboratory, a subsample of randomly selected indoor and outdoor-resting blood-fed females of anopheline mosquitoes was processed to determine the origin of blood meals (i.e., humans, bovines, ovines, chickens and equines) using the direct enzyme-linked immunosorbent assay (ELISA) method described by Beier et al. [21]. Genomic DNA was extracted from individual *An. gambiae* s.l. samples using the protocol of Collins et al. [22], and molecular identification was performed using the polymerase chain reaction (PCR) method described by Wilkins et al. [23].

### Data analysis

Indoor resting densities were calculated by dividing the number of *Anopheles* females collected from PSCs by the number of collection points (PSC rooms). The human blood index (HBI) was estimated as the ratio of the number of blood meals taken from humans to the total number of blood meals identified. The data were recorded in an Excel spreadsheet. R software (version 4.3.0) was used for all the statistical tests. The Student’s *t*-tests was used to compare means, and the Pearson’s chi-squared test was used to compare proportions. The significance level was set at 5%.

## Results

### Composition and distribution of the anopheline fauna

A total of 765 *Anopheles* female mosquitoes were collected during the entire study period. The collected samples consisted of 697 (91.1%) *An. gambiae* s.l., 63 (8.2%) *Anopheles rufipes*, 3 (0.4%) *An. funestus*, 1 (0.1%) *An. pharoensis* and 1 (0.1%) *Anopheles squamosus*. Mainland sites represented 23.7% (*n* = 181) of the collections, with 50.3% (*n *= 91) collected indoors and 49.7% (*n* = 90) collected outdoors. The abundance of *Anopheles* was significantly greater (*χ*^2^ = 19.09, *df* = 1, *P* = 1.25 × 10^−5^) on Wendaye Island (*n* = 584, 76.3%), with 70.9% (*n* = 414) collected indoors and 29.1% (*n* = 170) collected outdoors.

**Fig. 2:**
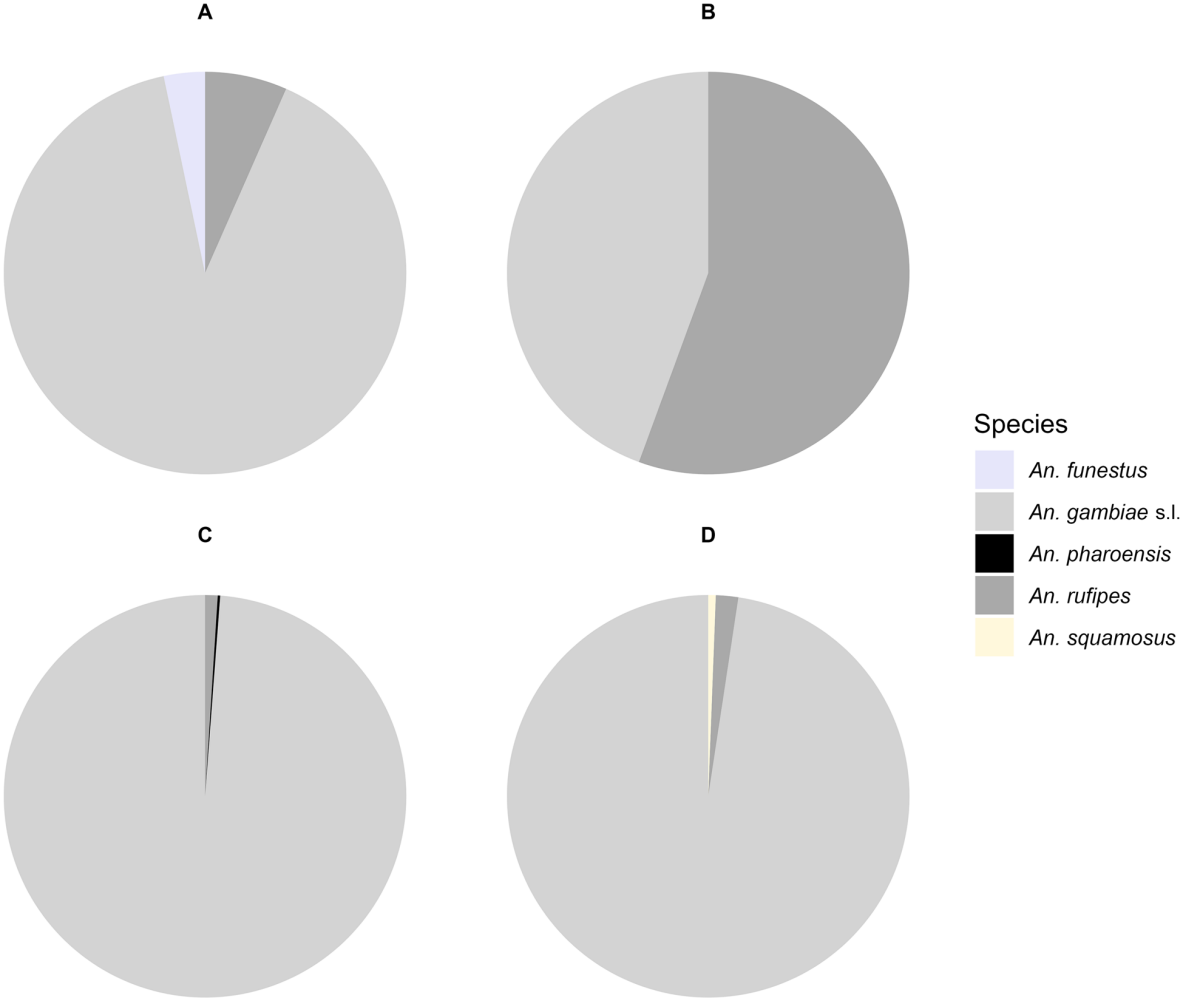
Species composition of resting *Anopheles* mosquitoes on the mainland and the island in south-western Senegal. **A** Indoor mainland. **B** Outdoor mainland. **C** Indoor island. **D** Outdoor island

Overall, *Anopheles*-specific diversity was low during the study period at both the mainland and island study sites (Fig. 2). Indoor resting females included *An. gambiae* s.l., *An. funestus* , *An. rufipes* and *An. pharoensis* specimens, whereas the outdoor collections consisted of *An. gambiae* s.l., *An. rufipes* and *An. squamosus*. At mainland sites, *An. gambiae* s.l. and *An. rufipes* were found indoors and outdoors, whereas *An. funestus* was only collected indoors. Similarly, at island sites, *An. gambiae* s.l. and *An. rufipes* were collected both indoors and outdoors, whereas *An. funestus* was absent during the study period. *Anopheles pharoensis* was collected indoors, and *An. squamosus* was collected outdoors, in low proportions (Fig. 2). As *An. gambiae* s.l. was predominant, subsequent comparisons focused on this species.

### *Anopheles gambiae* s.l. population dynamics and relative abundance

Endophilic and exophilic *An. gambiae* s.l. populations were dense at mainland sites in September of each study year. The mean relative abundances of the endophilic and exophilic populations were 23.2% and 40%, respectively, in 2020. Conversely, in 2021, the percentages of endophilic and exophilic females were 62.2% and 27.5%, respectively. Notably, *An. gambiae* s.l. females displayed an exophilic tendency during December 2020 and January 2021 (Table 1).

**Table 1:** Monthly variation in the relative abundance of *An. gambiae* s.l.

Months	Mainland				Island			
	PSC		Prokopack		PSC		Prokopack	
	*n*	%	*n*	%	*n*	%	*n*	%
September 2020	19	23.2	16	40.0	111	27.1	4	02.4
October 2020	00	00.0	00	00.0	181	44.3	21	12.7
November 2020	02	02.4	02	05.0	18	04.4	15	09.0
December 2020	00	00.0	06	15.0	14	03.4	30	18.1
January 2021	00	00.0	01	02.5	06	01.5	12	07.2
March 2021	01	01.2	00	00.0	04	01.0	06	03.6
May 2021	00	00.0	00	00.0	10	02.4	13	07.8
July 2021	02	02.4	02	05.0	21	05.1	30	18.1
September 2021	51	62.2	11	27.5	41	10.0	19	11.4
November 2021	07	08.5	02	05.0	03	00.7	16	09.6
Total	82		40		409		166	

PSC, pyrethrum spray catches

In comparison, the natural populations of *An. gambiae* s.l. displayed clear indoor resting behaviour in September (27.1%) and October (44.3%) of 2020. *Anopheles gambiae* s.l. outdoor resting behaviour was observed in October (12.7%) and December of 2020 (18.1%) and in July (18.1%) and September of 2021 (11.4%). Globally, *An. gambiae* s.l. females were more abundant in outdoor shelters and even more abundant in island populations regardless of the collection method (Table 1).

### Physiological state of *An. gambiae* s.l. resting females

The abdominal statuses of 697 *An. gambiae* s.l. resting females were determined. Among the 122 females collected from mainland sites, 75.6% and 57.5% of the samples collected indoor and outdoor, respectively, had recently fed. At the island sites, 63.3% of females collected indoors and 44.0% of females collected outdoors had recently fed (Table 2).

**Table 2:** Physiological state of *An. gambiae* s.l. females

Sites	Methods	Unfed		Fed		Half-gravid		Gravid		Total
		*n*	%	*n*	%	*n*	%	*n*	%	
Mainland	PSC	05	06.1	62	75.6	13	15.9	02	02.4	82
	Prokopack	07	17.5	23	57.5	04	10.0	06	15.0	40
Island	PSC	62	15.2	259	63.3	28	06.8	60	14.7	409
	Prokopack	36	21.7	73	44.0	23	13.8	34	20.5	166
Total		110		417		68		102		697

PSC, pyrethrum spray catches

### Resting behaviour and feeding pattern of *An. gambiae* s.l. females

#### Indoor resting densities

**Fig. 3:**
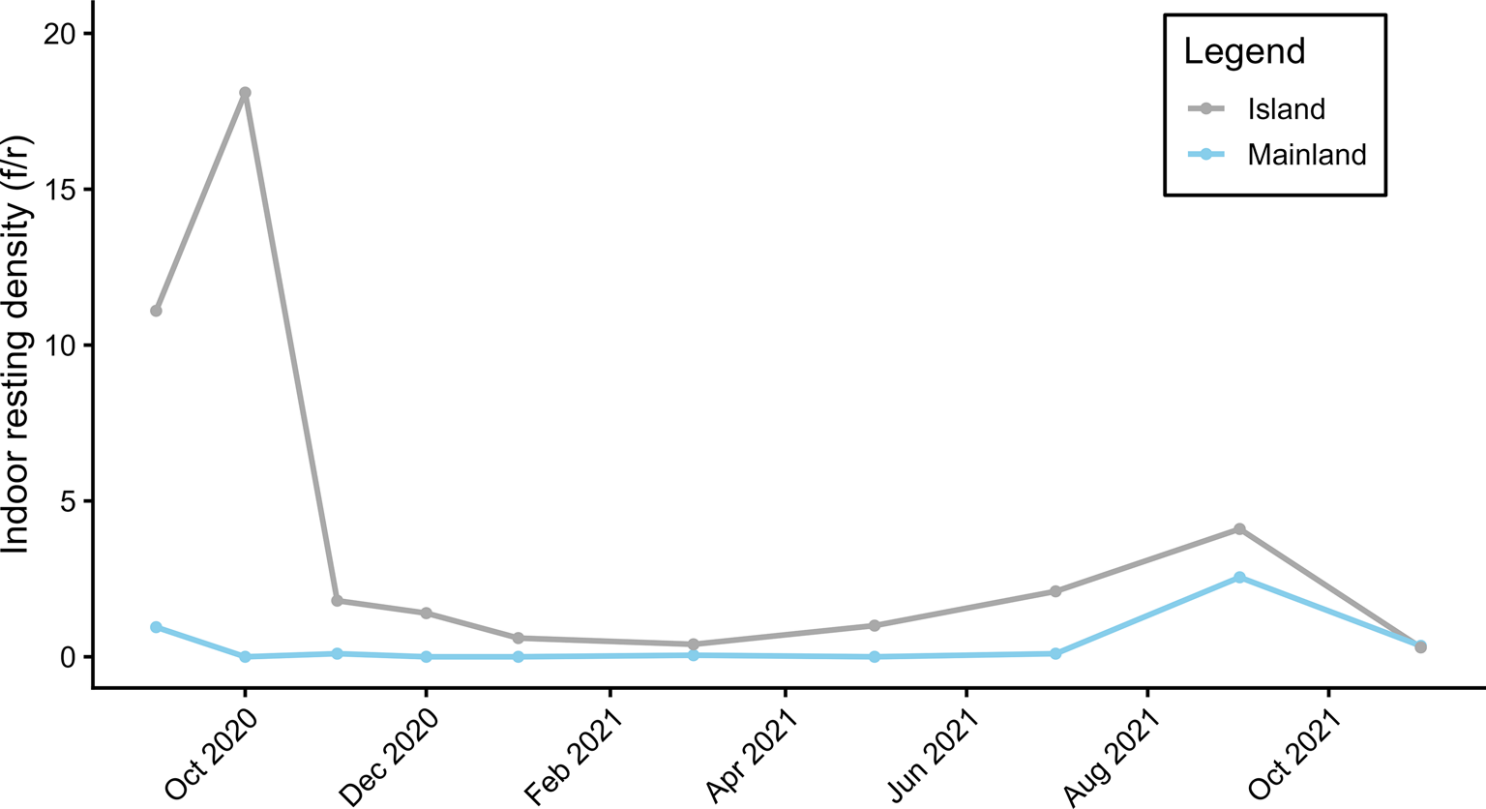
Indoor resting density of *An. gambiae* s.l. populations on the mainland and on the island in south-western Senegal from 2020 to 2021

The mean indoor resting density (IRD) of *An. gambiae* s.l. was significantly (*t* = −1.9461, *P* = 0.082) lower at mainland sites [0.41 females per room (f/r)] than at island sites (4.09 f/r). The highest IRDs were recorded in October 2020, with 18.1 f/r at island sites, and in September 2021, with 2.55 f/r at mainland sites (Fig. 3).

#### Trophic preferences

The blood meals of 87 *An. gambiae* s.l. females were tested, including 65 from indoor collections and 22 from outdoor shelters at mainland sites. For indoor resting females, 63 blood meals were successfully identified, including 48 samples from humans, 9 from bovines, 2 from sheep, 1 from chickens and 1 from mixed origin (sheep + chickens + equines). Of the 22 exophilic females tested, 21 blood meals were successfully identified, including 8 from humans, 6 from bovines and 2 from mixed origins 1 from humans + sheep + equines and 1 from humans + sheep + chickens + equines).

Meanwhile, a total of 330 blood meals from island sites were tested, including 257 from indoor resting females and 73 from outdoor resting females. Of the 231 successfully identified blood meals, 55 were from humans, 157 from bovines, 13 from sheep and 3 from mixed origins (human + sheep). Among the 73 outdoor resting females, 90 blood meals were successfully identified, with 16 from humans, 30 from bovines, 5 from sheep and 12 from mixed origins 2 from human + sheep, 1 from humans + bovines, 1 from humans + chickens, 1 from humans + sheep + equines and 7 from humans + sheep + chickens + equines) (Table 3).

**Table 3:** Origin of the blood meal of *An. gambiae* s.l. females

Sites				Vertebrate hosts					Mixed						HBI (%)
	T	I	N	H	B	S	C	E	HS	HB	HC	HSE	SCE	HSCE	
Mainland															
PSC	65	63	4	48	9	2	1	0	0	0	0	0	1	0	76.2
Prokopack	22	21	6	8	6	0	0	0	0	0	0	1	0	1	47.6
Total	87	84	10	56	15	2	1	0	0	0	0	1	1	1	69.0
Island															
PSC	257	231	29	55	157	13	0	0	3	0	0	0	0	0	25.1
Prokopack	73	90	10	16	30	5	0	0	2	1	1	1	0	7	31.1
Total	330	321	39	71	187	18	0	0	5	1	1	1	0	7	26.8
Total	417	405	49	127	202	20	1	0	5	1	1	2	1	8	35.5

T, tested; I, identified; N, negative; PSC, pyrethrum spray catches; H, human; B, bovine; S, sheep; C, chicken; E, equine; HBI, human blood index

#### Indoor and outdoor human blood index

At mainland sites, the human blood indices (HBIs) were 76.2% (48/63) and 47.6% (10/21) for endophilic and exophilic *An. gambiae* s.l. populations, respectively. The HBI was significantly greater in endophilic populations (*χ*^2^ = 4.42, *df* = 1, *P* = 0.035). Blood meals obtained from bovine hosts accounted for 14.3% (9/63) and 28.6% (6/21) of the endophilic and exophilic populations, respectively. Bovines were the second host of choice for *An. gambiae* s.l. resting females both indoors and outdoors. Conversely, the HBI at the island sites was 25.1% (58/231) for endophilic females and 31.1% (28/90) for outdoor resting females, with no significant difference between the two subpopulations (*χ*^2^ = 0.43, *df* = 1, *P* = 0.51). Therefore, bovines were the main blood source for both endophilic and exophilic *An. gambiae* s.l. populations at island sites constituted 68.0% (157/231) and 34.4% (31/90) of the blood sources, respectively.

The HBIs of *An. gambiae* s.l. significantly differed between mainland and island sites. The HBI was 25.1% at the island sites and 76.2% at the mainland sites for the indoor resting population. For exophilic populations, the HBI was 31.1% at island sites and 47.6% at mainland sites. The endophilic population at the mainland sites had a greater HBI than that at the island sites did (*χ*^2^ = 17.67, *df* = 1, *P* = 2.63 × 10^−5^). Conversely, the HBIs obtained for the exophilic populations did not significantly differ between the mainland and island sites (*χ*^2^ = 2.32, *df* = 1, *P* = 0.13) (Table 3).

### Molecular identification of *An. gambiae* sibling species

Subsamples of 324 *An. gambiae* s.l. specimens were identified at mainland (*n* = 75) and island (*n* = 249) sites by PCR. Molecular analysis identified *An. gambiae* s.s. (85.3%, *n* = 64), *An. arabiensis* (6.7%, *n* = 5), *An. coluzzii* (2.7%, *n* = 2) and *An. gambiae*/*An. coluzzii* hybrids (5.3%, n = 4) at mainland sites, whereas *An. gambiae* s.s. (77.1%, *n* = 192), *An. arabiensis* (20.9%, *n* = 52) and *An. gambiae*/*An. coluzzii* hybrids (2.0%, *n* = 5) were observed at the island sites. *Anopheles gambiae* s.s. was the most common species at both the mainland and island sites.

## Discussion

During this study, 5 of the 20 *Anopheles* species described in Senegal [6] were collected in the study area using two sampling methods targeting both indoor and outdoor resting mosquitoes. *Anopheles gambiae* s.s. was the predominant species, and its relative abundance displayed seasonal variation, following the rainfall regimes at the mainland and island sites. Compared with exophilic females, endophilic *An. gambiae* s.l. females exhibited a greater feeding preference for humans at mainland sites. Conversely, at the island sites, both endophilic and exophilic populations tended to be zoophilic.

The predominance of *An. gambiae* s.s. a primary malaria vector in several parts of Senegal is consistent with the findings of previous studies [24–26]. Its predominance could be explained by the existence of suitable ecological conditions for larval breeding sites and the survival of adult populations in the humid savannah and forest ecosystems in the southern part of Senegal. Moreover, the presence of multiple rice farms created favourable conditions for the proliferation of anopheline larval habitats, as reported elsewhere [1, 26–29]. The resting density of *An. gambiae* was greatest during the middle and end of the rainy season, between September and October, when permanent breeding sites were more common. The high mosquito densities during the dry season (December 2020 and May 2021) recorded in Wendaye are possibly attributable to the creation of ponds and paddles following the withdrawal of the watercourses crossing the island in their minor riverbeds during the dry season and/or the tidal rhythm [9]. The high densities of exophilic populations, especially at island sites at the beginning of the rainy season and during the dry season, could be explained by the existence of widespread favourable well-shaded outdoor resting sites offered by the abundant surrounding vegetation cover. Furthermore, the greater tendency towards the observed exophilic behaviour of *An. gambiae* s.l. could be an induced behavioural shift in response to the deployment of indoor insecticide-based vector control interventions, such as LLINs and IRS [30].

During the study, mosquito host preferences varied according to the sampling method and collection site. Some species show a highly specific trophic tendency, whereas others display more plasticity in terms of blood-feeding patterns, following the availability and accessibility of alternative hosts. *Anopheles gambiae* s.l. is generally known to have a stronger anthropophilic preference [24, 31–34].

On the mainland, *An. gambiae* s.l. endophilic females were highly anthropophilic, as observed by Faye et al. [35] in the middle Senegal River valley, as well as in other previous studies carried out under similar ecological conditions [9, 31, 36]. However, the exophilic tendency of *An. gambiae* s.l. populations provide a wider alternative blood source spectrum, which could explain the observed lower HBI and the greater tendency to feed on alternative animal hosts. This preference for alternative animal hosts may have resulted from the repellent effect of the LLINs used indoors, which could divert host-seeking females towards more available and accessible alternative animal hosts [8, 37].

At island sites, bovine hosts were the primary blood source for both the endophilic and the exophilic populations, which displayed lower anthropophilic preference. This preference could be explained by the predominance of bovine animals within the livestock population at this site. Indeed, the island is a restricted area and offered little space for keeping pets, which resulted in a high abundance of cattle and other animals living in the vicinity of households, and, consequently, the probable trophic deviation noted on Wendaye Island. Moreover, the widespread use of LLINs may also partially explain the observed tendency towards zoophagy [35]. These results are in line with previous observations on Reunion Island [38]. On Madagascar Island, Ravoahangimalala et al. [39] reported low HBIs, especially at the Ambohimahavelona (20%) and Kalandy (16%) sites. In Dogo (Ghana), very low HBIs were reported for *An. gambiae* s.l. populations [40]. Observations of the trophic preferences of endophilic [41] and exophilic *An. gambiae* s.l. populations revealed that the human host became increasingly inaccessible under natural conditions and that several vector species are diverted to alternative domestic animals. However, multiple animals are kept close to human habitations, and zoophagic species can maintain residual transmission when gaps in protection occur. Further investigations are needed to address opportunistic tendencies.

The results of this study provide key information on the predominant species in the area while revealing different feeding and resting patterns between mainland and island sites. During this study, *An. gambiae* s.l. resting females were found both indoors and outdoors. Natural *An. gambiae* s.l. populations displayed variable trophic preferences depending on the availability and accessibility of different hosts. *Anopheles gambiae* s.l. remained highly polyphagous, although endophilic females fed preferentially on humans at mainland sites. Bovines were the preferred host for zoophagous females at both the mainland and island sites. However, the persistence of competent malaria vectors in the area should be considered residual outdoor malaria transmission. Evidence on vector behaviours will enable control programmes to select appropriate interventions. This study serves as a starting point for providing preliminary evidence on key vector feeding and resting behaviours to optimize and complement current and future control strategies.

In terms of limitations, evaluating the involvement in malaria transmission and assessing the contribution of exophilic *An. gambiae* s.l. populations in malaria transmission would be interesting. In the south of the country, changes in resting behaviour need to be closely monitored to implement interventions complementary to LLINs and IRS that will target outdoor resting vectors. The sterile insect technique, which involves altering the fertility of an insect to control its population, would be an effective tool for combating exophilic *An. gambiae* s.l. populations in the area.

## Conclusions

This study was conducted in an area with a low malaria incidence in south-western Senegal, where few areas of residual transmission provide preliminary information about the feeding and resting behaviours of *An. gambiae* s.l. Preliminary results revealed variable dynamics, with marked seasonal fluctuations in indoor and outdoor resting *An. gambiae* s.s., the main malaria vector that feeds both on humans and animals. However, the observed high HBIs at mainland sites contrasts with the high level of zoophagic rates observed at island sites. The persistence of competent malaria vectors in the study area, which is not impacted by indoor-based vector control interventions, placed the human population at risk of outdoor residual malaria transmission across the entire study area. These preliminary results provide basic information for understanding *An. gambiae* s.s. feeding and resting behaviours, which are key for evidence-based interventions to control the southern Senegal zoophilic and exophilic malaria vector populations and eliminate malaria in this region.

## Data Availability

All the data are available in the main manuscript text.

## Abbreviations

DNA: Deoxyribonucleic acid
ELISA: Enzyme-linked immunosorbent assay
f/r: Females per room
HBI: Human blood index
IRD: Indoor resting density
IRS: Indoor residual spraying
LEVP: Laboratoire d'Ecologie Vectorielle et Parasitaire
LLINs: Long-lasting-insecticide-treated nets
PCR: Polymerase chain reaction
PSC: Pyrethrum spray catches
